# Utilization of community health workers for malaria treatment: results from a three-year panel study in the districts of Kaya and Zorgho, Burkina Faso

**DOI:** 10.1186/s12936-015-0591-9

**Published:** 2015-02-13

**Authors:** Thomas Druetz, Valéry Ridde, Seni Kouanda, Antarou Ly, Souleymane Diabaté, Slim Haddad

**Affiliations:** School of Public Health, University of Montreal, 7101 avenue du Parc, Montreal, QC H3N 1X9 Canada; University of Montreal Hospital Research Centre, 850 rue Saint-Denis, Montreal, QC H2X 0A9 Canada; Biomedical and Public Health Department, Institut de Recherche en Sciences de la Santé, Ouagadougou, 03 BP 7192 Burkina Faso; Faculty of Medicine, Laval University, 1050 avenue de la Médecine, Québec, QC G1V 0A6 Canada; Laval University Medical Research Center (CHUQ), Saint-Sacrement Hospital, 1050, chemin Sainte-Foy, Québec, QC G1S 4L8 Canada

**Keywords:** Community case management, Community health worker, Malaria, Health-seeking behavior, Burkina Faso, Sub-Saharan Africa, Panel study, Evaluation

## Abstract

**Background:**

Malaria is holo-endemic in Burkina Faso and causes approximately 40,000 deaths every year. In 2010, health authorities scaled up community case management of malaria with artemisinin-based combination therapy. Previous trials and pilot project evaluations have shown that this strategy may be feasible, acceptable, and effective under controlled implementation conditions. However, little is known about its effectiveness or feasibility/acceptability under real-world conditions of implementation at national scale.

**Methods:**

A panel study was conducted in two health districts of Burkina Faso, Kaya and Zorgho. Three rounds of surveys were conducted during the peak malaria-transmission season (in August 2011, 2012 and 2013) in a panel of 2,232 randomly selected households. All sickness episodes in children under five and associated health-seeking practices were documented. Community health worker (CHW) treatment coverage was evaluated and the determinants of consulting a CHW were analysed using multi-level logistic regression.

**Results:**

In urban areas, less than 1% of sick children consulted a CHW, compared to 1%–9% in rural areas. Gaps remained between intentions and actual practices in treatment-seeking behaviour. In 2013, the most frequent reasons for not consulting the CHW were: the fact of not knowing him/her (78% in urban areas; 33% in rural areas); preferring the health centre (23% and 45%, respectively); and drug stock-outs (2% and 12%, respectively). The odds of visiting a CHW in rural areas significantly increased with the distance to the nearest health centre and if the household had been visited by a CHW during the previous three months.

**Conclusions:**

This study shows that CHWs are rarely used in Burkina Faso to treat malaria in children. Issues of implementation fidelity, a lack of adaptation to the local context and problems of acceptability/feasibility might have undermined the effectiveness of community case management of malaria. While some suggest extending this strategy in urban areas, total absence of CHW services uptake in these areas suggest that caution is required. Even in rural areas, treatment coverage by CHWs was considerably less than that reported by previous trials and pilot projects. This study confirms the necessity of evaluating public health interventions under real-world conditions of implementation.

**Electronic supplementary material:**

The online version of this article (doi:10.1186/s12936-015-0591-9) contains supplementary material, which is available to authorized users.

## Background

Community case management of malaria (CCMm, formerly designated as home management of malaria) consists in treating febrile individuals with pre-packaged anti-malarial drugs distributed by members of the community, often designated as community health workers (CHWs) [1]. CCMm is a strategy gaining popularity in sub-Saharan Africa (SSA), where malaria remains one of the main killers – in 2010, approximately 1.14 million deaths were attributed to malaria in SSA, 700,000 of them being children under five [2]. Providing effective malaria treatments within 24 hours of fever onset remains a key challenge in the fight against malaria in SSA [3,4].

Trials and pilot project evaluations have shown that CCMm with artemisinin-based combination therapy (ACT) may be feasible, acceptable and effective for treating simple malaria cases [5-12]. It may also contribute to lessening workload at primary health centres [13], increasing promptness of treatment [14] and reducing health inequities [3]. These promising results have generated considerable enthusiasm, and studies are in progress to evaluate the potential benefits of combining CCMm with other anti-malaria interventions (e.g. intermittent preventive treatment [4,15-17]) or of implementing integrated community management of malaria, pneumonia, and diarrhoea [18,19]. At the same time, the use of rapid diagnostic tests by CHWs has been evaluated as successful and is becoming a key component in CCMm strategy [20-22]. Initially planned to be implemented in remote areas with difficult access to health centres, a recent study has also suggested that CCMm may be relevant in urban areas of highly malaria-endemic countries [12].

However, some evidence gaps remain. A recent meta-analysis pointed out the lack of evidence regarding CCMm impacts on mortality [23]. Moreover, most evaluations of CCMm efficacy took place under favourable conditions of randomized controlled trials or pilot projects. Therefore, little is known about its effectiveness, feasibility and acceptability under real-world conditions of implementation [24-26], despite the fact that several implementation barriers have been identified – drug stock-outs, referral completion, adherence to treatment guidelines, programme sustainability [27-30]. Finally, while it is argued that CCMm reduces the monetary and geographical barriers that impede individuals’ seeking treatment, few studies have evaluated the extent to which CHWs are used in an uncontrolled context of nation-wide CCMm, or how treatment coverage by CHWs varies with time [25,31].

Malaria is holo-endemic in Burkina Faso and causes the deaths of ~40,000 individuals every year [2]. In 2010, health authorities scaled up CCMm to the national level without waiting for complete evaluations from three pilot projects [32]. The intention is to examine health-seeking behaviour in the context of CCMm and to advise health authorities, in light of these findings, on its effectiveness in the Burkinabé context. The objectives of this panel study are to (1) assess the extent to which CHWs are used by caregivers of sick children over a three-year period after the introduction of CCMm, and (2) determine what influences their health-seeking practices.

## Methods

### CCMm implementation and study context

Burkina Faso introduced CCMm with ACT in 2010. The five-year budget dedicated to CCMm is 5.8 million Euros and is part of a larger 63 million Euro grant received from the *Global Fund to Fight AIDS, Tuberculosis and Malaria* [33]. In every village, a CHW was recruited and trained to administer treatments to sick individuals with reported fever. Each urban health centre also recruited a CHW to implement CCMm in urban sectors. CHWs were provided with some resources (ACT, bicycles, job aids) and received a monthly compensation of ~10 USD. Visits to CHWs have been free-of-charge, but medication costs 0.2–0.6 USD depending on the individual’s age. CHWs refer individuals with danger signs (convulsions, unconsciousness, difficulty to drink or persistent vomiting) and pregnant women to the nearest health centre [34]. CHWs also conduct home visits and hold awareness sessions to disseminate prevention information. In 2012, because of nation-wide issues with ACT supplies [32,35], CCMm was downgraded to low priority. The implementation fidelity of the programme was measured prior to this study (June-August 2011) in the two districts under investigation – Kaya and Zorgho – and results have been published elsewhere [36]. Some issues concerning drug supply, CHWs remuneration, and the involvement of actors from civil society have been observed [36,37]. The programme was implemented at national scale without evidence to support its feasibility and acceptability in the Burkinabé context – indeed CCMm was scaled up before the evaluation of pilot projects could be completed.

In Kaya, one of the three pilot sites, CCMm was initiated in the beginning of 2010, while in Zorgho it was introduced approximately nine months later. Two additional interventions were in progress in the district of Kaya prior to this study and might have influenced treatment-seeking behaviour. The first, introduced by the Bill & Melinda Gates Foundation in October 2010, consisted in using CHWs to manage childhood illnesses (malaria and diarrhoea); the theory of this intervention is very similar to CCMm. The second intervention was implemented in July 2011 by Save the Children (financed by European Commission Humanitarian Aid) and consisted in removing health centres user fees for all children under five. Previous studies conducted in Burkina Faso and elsewhere showed that abolishing user fees significantly increased health centre services uptake by sick children and reduced health inequities [38,39].

### Study design

This is a household panel study conducted in two health districts of Burkina Faso, Kaya and Zorgho (Table 1). Both districts are situated in areas where malaria is holo-endemic and transmission occurs during or briefly after a prolonged rainfall season, which lasts every year from June until November. The Kaya site was selected first due to the existence of a health and demographic surveillance system – Kaya HDSS [40] – that lends itself to the study. To increase internal validity, a comparison district (Zorgho) that was not a pilot site and was not contaminated by concomitant interventions was selected.

**Table 1:** **Characteristics of study sites**

	**Kaya district**	**Zorgho district**
Number of malaria infections per inhabitant per year	0.25	0.38
Annual rainfall	506 mm	661 mm
Households below the poverty line	44 %	41 %
Main spoken language	90 % (Mooré)	89 % (Mooré)
Population	500 208	352 003
Distance from capital city	98 km	103 km
Number of primary health centres	48	44

Table adapted from Ridde et al. [36].

In both districts, a household panel study was conducted from 2011 to 2013. The study area included 15 villages and two urban sectors (in Kaya) and 17 villages and one urban sector (in Zorgho). All villages are located within a 20-km radius of the cities of Kaya or Zorgho. A two-step sampling method was used to select households. First, a baseline census of all households in the study area was performed. A random selection was then carried out involving 3,002 individual households from among those inventoried (2,004 in Kaya and 998 in Zorgho – the panel size being double in Kaya for the purposes of other analyses). The random sampling was stratified to ensure an equal number of households in urban and rural areas. Among the 3,002 households, only those with children under 60 months of age were enrolled in the panel (N = 2,237).

All households agreed to participate in the study. They were all visited once a year during the peak malaria-transmission season (“total population design” [41]), which starts 30 days after 100 mm of rainfall [42]. Three rounds of surveys were conducted: in August 2011, 2012, and 2013. All households enrolled at baseline were followed in subsequent years. Those who had migrated out of the study area or could not be located were replaced by randomly selected households from the same district and area (rural or urban).

### Data collection

Data were collected through standardized household surveys based on the Malaria Indicator Surveys [43]. These were administered by 12 research assistants, who digitally encoded the data using iPAQ personal digital assistants (PDAs). Assistants received a five-day training before each round of surveys; most of them were employed for the full three years of the study.

Three types of questionnaires were administered. The first documented household composition, its assets (livestock, communication, transportation, energy), details regarding housing and crops, and the members’ activities. It was only administered once, when the household entered the panel. Each household was geo-referenced using a global positioning system (GPS). The second questionnaire was administered every year and concerned caregivers’ attitudes and practices towards malaria. It explored how they would intend to seek treatment for a febrile child, their reasons for not preferring to consult a CHW, and the number of times the household had been recently visited by a CHW (recall period: three months). The final questionnaire investigated recent sickness episodes in children under five. A sick child was defined as a child who had been sick recently, as declared by the caregiver (recall period: two weeks). Characteristics of episodes were documented, such as duration, presence of danger signs or other symptoms (fever, diarrhoea, cough), and treatment-seeking actions. Danger signs were defined using WHO classification and included not drinking/breastfeeding, persistent vomiting, lethargy, and convulsions [44]. Generally respondents were (one of) the mother(s) in the household.

Rainfall measures were obtained from meteorological centres in Kaya and Zorgho. Health centres in the study area were geo-referenced, and geodetic ellipsoidal distances between households and health centres were calculated using the *Geodist* add-on for Stata®. To confirm ages, children’s birthdates were extracted from vaccination booklets.

### Data analysis

The main outcome of this study was the source of the first treatment administered to sick children. Answers fell into five mutually exclusive categories: CHW, health centre, self-medication, traditional healer, or no treatment. While private healthcare was not an available source of first treatment in the study area, the category “self-medication” included caregivers who bought treatments at drug shops. The focus was on the first treatment because the intent of CCMm is for CHWs to be the first line of consultation for sick individuals – the target being that, by 2013, CHWs would be administering treatments to 80% of all simple malaria cases [33]. In addition, few children (10%) received more than one treatment. The study also had two secondary outcomes: (1) the caregivers’ reported intention for treatment in the hypothetical case of a febrile child and (2) the reasons for not preferring the CHW, if applicable.

Analysis of treatment-seeking behaviour examined variations between the districts, areas (rural or urban), and years of study. Caregivers’ intention for treatment was compared with actual consultations for recently sick children. Reasons for not having the intention to visit a CHW were explored – as results were consistent over the three years, only data from the 2013 survey were presented.

Logistic regression was used to identify the factors associated with caregivers’ practice of bringing a sick child to a CHW. The main outcome variable was re-coded ‘yes’ if a sick child had visited a CHW and ‘no’ if not. Analysis was conducted in a sub-sample; only rural households were retained because of the quasi-absence of visits to CHWs in urban areas. Independent variables were identified by examining the study context, the logic model of the programme, and the literature on treatment-seeking behaviour [31,45-48]. Children-level independent variables were age, sex, and presence of symptoms during the episode. Household-level covariates included family size, polygamy status, ownership of lands or cattle, distance to the nearest health centre, and the fact of having been recently visited by a CHW. Land property and cattle ownership were used as proxies for wealth; they had been previously identified as major determinants of socioeconomic status in rural areas of Burkina Faso [49,50]. The year of the study and the district were also included in the model. Two other confounding variables mentioned in the literature, i.e., the occupation of heads of household and mothers’ education level, were discarded because of their undiscriminating nature – 92% of heads were farmers and 96% of mothers were illiterate.

Variance inflation factors were computed to detect multicollinearity between the variables using the *Collin* add-on for Stata®. Interactions between the district and the year, on one hand, and each of the 10 other variables, on the other, were examined using likelihood ratio tests and assessing coefficient changes. Independent variables were entered in the fixed part of a three-level (child, household, village) logistic model to take into account the hierarchical structure of the data. Predictive probabilities of visiting a CHW were computed after the model fit was found to be acceptable. All analyses were performed using Stata version 13 (StataCorp LP, College Station, TX).

### Ethical approval

Ethical approval was obtained from the research ethics committee of the University of Montreal Hospital Research Centre in Canada and Burkina Faso’s health research committee. Written consent was obtained each year from the respondent (usually the mother) of every household. Ethical procedures were derived from the Malaria Indicator Survey instruments. Children with danger signs were immediately referred to the health center. Households could stop participating in the survey or choose not to answer a question at any time. The confidentiality of their answers was guaranteed.

## Results

The main characteristics of children enrolled in the cohort and their households are detailed in Table 2. The number of children who had been sick in the previous two weeks reached 706 in 2011 (24.6%), 792 in 2012 (25.4%), and 830 in 2013 (26.7%). Few of these children (respectively 11%, 13%, and 7%) received treatment from more than one source.

**Table 2:** **Main characteristics of the panel at baseline (2011)**

**Children**	**Kaya**	**Zorgho**
Number	1,778	1,092
Age in months (median, iqr)	30 (28)	30 (27)
Female	884 (50%)	552 (51%)
Sick over the past 2 weeks	544 (31%)	162 (15%)
Slept under a bed net the night before	1,147 (66%)	560 (52%)
**Households**		
Number	1207	591
In urban areas	522 (43%)	240 (41%)
Polygamous	433 (36%)	259 (44%)
3-years follow-up	1,096 (91%)	540 (91%)
Head is a farmer	869 (73%)	439 (74%)
Illiterate mother†	810 (88%)	457 (84%)
Number of bed nets (median, iqr)	2 (1)	3 (3)
Household size (median, iqr)	7 (4)	9 (7)

Iqr interquartile range † some data are missing.

### Descriptive analysis of treatment-seeking behaviour

**Figure 1:**
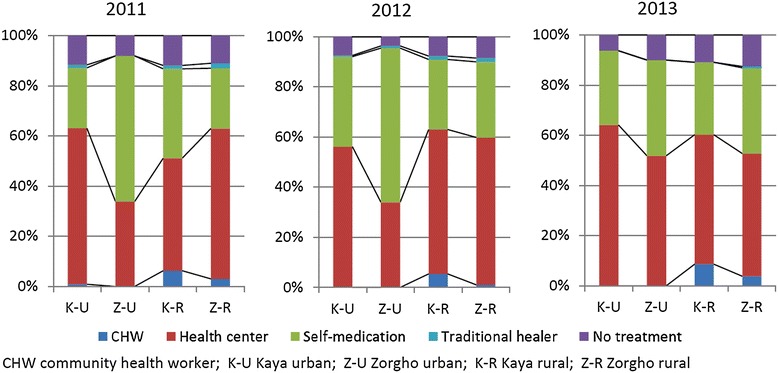
**First treatment-seeking action for sick children under five years of age.**

In rural areas of both sites and in the urban area of Kaya, the health centre was the most frequent source of first treatment (Figure 1), before self-medication. In the urban area of Zorgho, self-medication was more popular than consulting the health centre in 2011 and 2012, but the situation was reversed in 2013. In all instances the absence of treatment came in third position. Between 4% and 13% of sick children received no treatment, depending on the site area and the year.

In urban areas, less than 1% of sick children visited a CHW as the first source of treatment. In rural areas, this proportion varied between 1% and 9% and was slightly higher in Kaya than in Zorgho. The likelihood of visiting a CHW did not improve with time and was similar for sick children overall, sick children with a reported fever, and sick children without danger signs (see Additional file 1).

**Figure 2:**
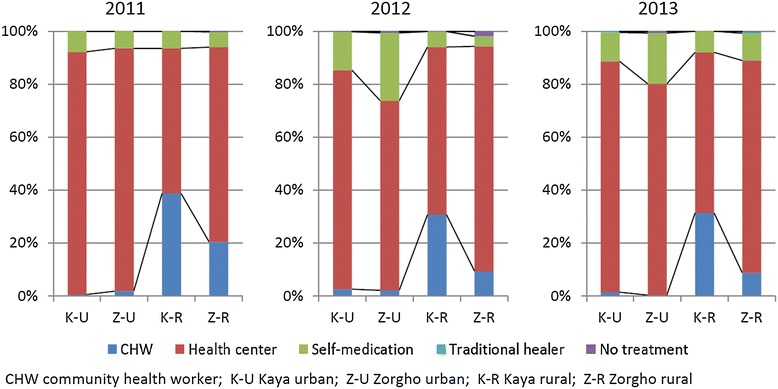
**First treatment-seeking action intended by caregivers of a febrile child.**

There were differences between the source of treatment actually selected for sick children and the source of treatment that caregivers reportedly intended to choose for a febrile child (Figure 2). The proportion of children without treatment or treated by self-medication was higher than the proportion of caregivers who had intended to choose these options. Conversely, intention to visit health centres (in all areas) and CHWs (in rural areas) exceeded the actual proportion of sick children brought to these providers. There were also some congruencies. For example, both the intention to visit and the actual use of traditional healers never exceeded 2%, regardless of the area or the year. Similarly, in urban areas, both the intended and actual use of CHWs were hardly ever reported.

**Figure 3:**
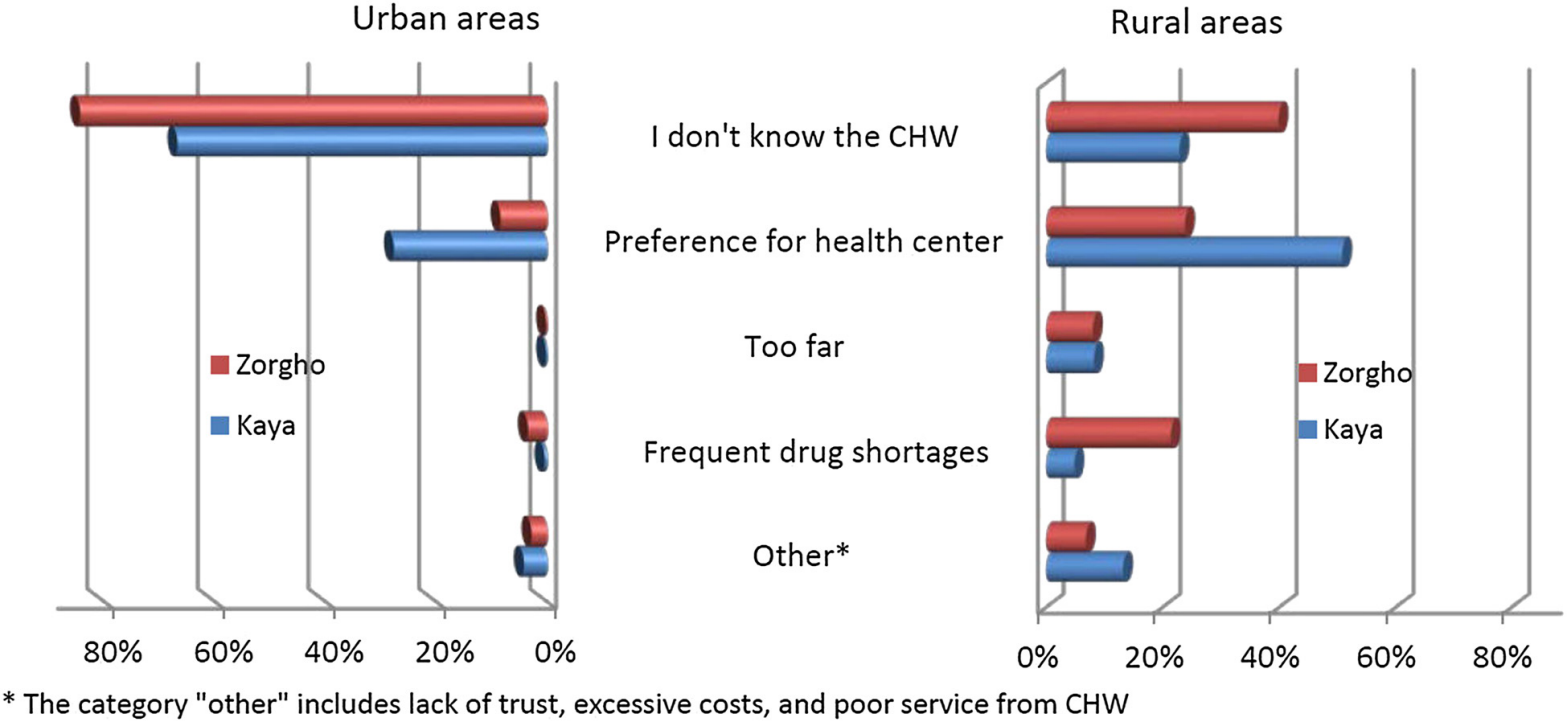
**Caregivers’ reason for not intending to consult the community-health worker as first treatment-seeking action.**

In 2013, 1,781 caregivers (86%) stated they intended to choose a source of treatment other than the CHW. When asked why, they identified a number of possible reasons for not choosing the CHW. In urban areas, not knowing about the CHW was cited by 78% of caregivers. The second most common answer was preference for the health centre (23%) (Figure 3). Other factors were rarely mentioned. In rural areas, the most frequent reasons were preference for health centres (45%), not knowing the CHW (33%), CHWs’ frequent drug stock-outs (12%), and distance to the CHW (8%). Distrust in CHWs, excessive costs, or poor service were hardly ever mentioned.

### Determinants of consulting a CHW in rural areas

Analysis of determinants of health-seeking behaviour was performed on rural households only. The characteristics of sick children (for the three years combined) are detailed in Table 3. Some differences were statistically significant (p < 0.05) between the two districts. Sick children were more frequent in Kaya and they presented danger signs or symptoms of cough/diarrhea more often than in Zorgho. More sickness episodes were still ongoing at the time of the survey in Zorgho than in Kaya.

**Table 3:** **Descriptive statistics of children <5 years who had recently been sick (rural areas)**

**Characteristics of sick children**	**Kaya**	**Zorgho**	**Difference Zorgho - Kaya**
	**(n = 960)**	**(n = 483)**	
Girls	456 (47%)	244 (51%)	+4%
Age in months (median, iqr)	30 (26)	31 (25)	−1
With reported fever	857 (89%)	421 (87%)	−2%
With reported danger signs	244 (25%)	98 (20%)	−5%*
With reported cough/diarrhea	246 (26%)	54 (11%)	−15%***
Sickness episode still ongoing at the time of survey^†^	222 (24%)	137 (32%)	+8%**
Sick children among population	28%	21%	−7%***

Heterogeneity tests performed: Pearson χ2 or analysis of variance.

Iqr interquartile range; ^†^6% of data are missing; *p <0.05 **p <0.01 ***p <0.001.

A multilevel logistic regression was used to identify the determinants of caregivers’ practice of bringing their sick child to a CHW (Table 4). All significant associations were in the anticipated direction. The use of CHWs significantly increased with the distance to the nearest health centre and if the household had been recently visited by a CHW. The odds of visiting a CHW were higher in Kaya than in Zorgho and rose between 2012 (year of a nation-wide ACT shortage) and 2013. Intra-class correlation coefficients show that 76% (1 - (0.139/0.583)) of the unexplained variance was attributable to the household level, and 24% was attributable to the village level. No unexplained variance was attributable to the child level.

**Table 4:** **Multilevel logistic model of determinants of bringing sick children to a CHW**

**Fixed effects (reference category)**	**Odds ratio**	**95% CI**
Year (2012)¶		
2011	1.77	0.74 - 4.25
2013	2.73*	1.22 - 6.15
District (Zorgho)		
Kaya	5.7*	1.39 – 23.47
Distance to the nearest health centre (<2.5 km)		
2.5 km ≤ x > 5 km	7.16**	1.99 - 25.69
≥5 km	14.04***	2.97 - 66.51
Home visited by a CHW during the last 3 months (no)		
Yes	6.08*	1.51 - 24.40
Household size (less than 5)		
5 or more	2.05	0.59 - 7.07
Land owner (no)		
Yes	1.29	0.48 - 3.48
Possession of cattle (no)		
Yes	1.26	0.55 - 2.87
Polygamous household (no)		
Yes	1.17	0.53 - 2.62
Presence of danger signs (no)		
Yes	0.71	0.32 - 1.58
Presence of cough and/or diarrhea (no)		
Yes	1.27	0.57 - 2.84
Child sex (male)		
Female	0.87	0.44 - 1.71
Child age (<1 year)		
1-2	0.96	0.30 - 3.06
2-3	0.81	0.25 - 2.64
3-4	1.94	0.61 - 6.12
4-5	2.19	0.63 - 7.58
**Random effects** (level)		
ICC (village)	0.139	
ICC (village + household)	0.583	
ICC (village + household + child)	0.583	
Likelihood ratio test between single- and multi-level models: χ^2^ = 34.52***		

ICC Intra-class correlation coefficient; *p-value <0.05 **p-value <0.01. ***p-value <0.001.

¶2012 was used as the reference category because CCMm was nearly halted that year.

All interactions turned out to be non-significant (p > 0.1). Variance inflation factors never exceeded 1.30, which denotes absence of multicollinearity, and the Wald chi-square test indicated a satisfactory goodness-of-fit of the model (p < 0.05). Predicted probabilities of consulting a CHW were computed according to the district, the distance to the nearest health centre, and the fact of being recently visited by a CHW (Table 5). The highest probability reached 28% and was predicted in Kaya households recently visited by a CHW and far (>5 km) from a health centre. The smallest predicted probability (households in Zorgho close to health centres and not visited) was 0.05%.

**Table 5:** Predicted probabilities of consulting a CHW*

	**Kaya**	**Zorgho**
Households recently visited by a CHW		
no	0.008	0.002
yes	0.047	0.009
Distance to the nearest health centre		
<2.5 km	0.003	0.0006
2.5 - 5 km	0.021	0.005
>5 km	0.04	0.009

*adjusted for year, age, sex, sickness symptoms, household size and possessions, polygamous status.

## Discussion

This study examined the uptake of CHW services and its determinants during a three-year period after the introduction of CCMm in Burkina Faso. This country has a long history of using CHWs as providers of primary care, but with mixed results [51]. In 2010 it implemented CCMm at the national level, under routine conditions. Results from this study differ considerably from evaluations of CCMm interventions implemented under favourable conditions (controlled trials or pilot projects).

While some authors have argued that CCMm may be relevant in urban areas of SSA [12,25], results presented here indicate the opposite. Treatment coverage by CHWs was less than 1%; in comparison, Akweongo *et al.* reported an average of 40% from pilot projects in urban areas [12]. Even when considering caregivers’ intentions, less than 3% of them opted for the CHW. Several factors could explain this lack of uptake. CHWs’ involvement as well as their supervision might have been poorer in urban health centres because nurses have a lighter workload than in rural health centres [52,53], and so might be less inclined to take on task-shifting. Lack of information is another potential factor, since three years after the introduction of CCMm a majority (65%–85%) of urban caregivers still reported not knowing the CHW. Finally, in urban areas, it is common that people do not know where CHWs live, but instead see them occasionally at the health centre. The extension of CCMm into urban areas, which was not expressly planned in Burkina Faso but exists *de facto* [33], should be called into question. That being said, malaria transmission is certainly not insignificant in urban areas – prevalence reached 11% in the urban households of the panel. However, other therapeutic options should be considered.

In rural areas, the extent to which caregivers of sick children visited CHWs was smaller than expected, as treatment coverage by CHWs ranged between 1% and 9%; in comparison, pilot projects or controlled studies reported treatment coverage between 29% and 79% [6,14,31,54]. Caregivers’ intention to consult a CHW varied between 9% and 39%, which confirms that gaps persist between intention and practice in treatment-seeking behaviour [12,54]. Among the reasons cited for not visiting the CHW, preference for the health centre was mentioned twice as often in Kaya (51%) than in Zorgho (24%), which is consistent with the fact that healthcare is free-of-charge in Kaya. As in urban areas, not knowing the CHW was commonly cited (23% in Kaya, 40% in Zorgho), which suggests shortcomings in the programme’s communication strategy. While the lack of success of CCMm needs to be explored further, the analysis of determinants raises several points to be considered.

The probability of consulting a CHW for a sick child was significantly higher in Kaya than in Zorgho, despite the fact that healthcare for children is free in Kaya health centres. Several elements could explain this incongruous result. First, CCMm was a pilot project in Kaya, started before the national scale-up, and was more established than in Zorgho. Second, the presence of another community case management intervention (implemented by the Bill & Melinda Gates Foundation) in Kaya might have increased the population’s knowledge of and proclivity to consult CHWs. Finally, Zorgho faced more implementation challenges than did Kaya: CHW remuneration was more irregular; nurses did not participate in supervising CHWs; and ACT shortages were more severe. Further qualitative interventional research on implementation issues would be required to determine how CCMm programmes could be made more effective [55]. The proportion of sick children brought to health centers for first treatment was higher than was found in a previous study conducted in Burkina Faso (~57% vs. ~20%) [56]. Several factors might explain this high use of health centers: (1) in Kaya, as mentioned above, consultations and treatments have been free-of-charge at health centers for children since 2011; (2) ACTs are more expensive and harder to find in drug shops because of health regulations; (3) there has been a constant increase in the number (and use) of primary healthcare centers in Burkina Faso over the last few years [57].

There was a significantly higher proportion of sickness episodes among children in Kaya than among those in Zorgho, and the presence of danger signs or other symptoms was reported more often. Higher self-reported morbidity has already been observed in studies after removal of user fees and may be explained by caregivers’ improved knowledge due to increased contact with health centres [58].

The distance to the health centre was significantly associated with the probability of consulting a CHW, which corroborates other studies’ findings [12,31,45,54]. CCMm relevance in remote areas is supported by the fact that distance to health centres remains the most important barrier to care in rural Burkina Faso once user fees have been reduced [59]. These results are consistent with the argument that CCMm is effective in reaching remote communities and can reduce geographical inequities in health [60]. However, it seems appropriate to reformulate the statement by Akweongo *et al.* [12]: if CHWs are consulted, it is not because of their proximity to the household, but rather because health centres are far. In this study, if both the health centre and the CHW were close (within 5 km), the latter was hardly ever visited.

A significantly higher treatment coverage by CHWs was observed in the households they had visited in the previous three months, but this concerned only 5% of the panel. The low number of visits paid by CHWs might be explained by their heavy farming workload during the rainy season and their modest remuneration – a recent study has shown that these factors reduced their performance [37]. A prolonged absence of visits or information sessions may be interpreted by the population as an interruption of CCMm; indeed, CHW activities have been on-and-off since the start of community-based programmes in the 1980s, a situation common in West Africa [27,61]. CHW performance is hard to achieve and to maintain; this constitutes one of the biggest challenges for CCMm strategies [62]. Previous studies suggest that CCMm in Burkina Faso has faced some of the most common obstacles to performance: insufficient remuneration, drug stock-outs, lack of CHW training or refresher courses, insufficient supervision, and poor community participation [32,36,37].

From 2011, treatment coverage by CHWs never exceeded 10% in the rural population of the panel. The programme remained mostly unchanged during the study period, with the exception that CCMm was seriously curtailed and even halted in 2012 due to implementation problems (drug stock-outs). This situation might have undermined CHW credibility and dissuaded some villagers from consulting CHWs even after their stocks were replenished. Widespread ACT shortage is a common issue in SSA [27,63] and requires that CCMm include measures to strengthen countries’ health systems [47]. With limited capacities or room to manoeuver, Burkinabé health authorities might not have been able to effectively monitor CCMm activities and/or to react to the challenges that arose. It is noteworthy that, despite its ambitious objective (CHWs to manage 80% of all simple malaria cases), CCMm in Burkina Faso was granted an annual budget of only 1.16 million Euros. This represents less than 10% of the overall funding to scale up interventions against malaria; the majority of the budget (74%) was allocated to another intervention, a bed net distribution campaign [33]. This seems to confirm a statement by Kamal-Yanni *et al.* that there has been “no serious attempt to globalize investment in CHWs as a strategy to combat malaria” [64].

Recently, community health has received considerable attention in Burkina Faso. Authorities have piloted several evaluations of community-based interventions and have started to introduce integrated community case management – not in the study area, but elsewhere in the country [65,66]. Arguably, scaling-up this strategy should not be a priority since (1) there is a lack of evidence supporting its impacts and effectiveness in SSA [67], and (2) the results presented here suggest that CCMm encountered severe difficulties with regard to implementation, feasibility or acceptability in the local context. These issues should be settled before extending CCMm to other diseases. The health system and peripheral health centres need to be strengthened, if they are to contribute adequately to improving child survival [68]. At the same time, the population-based approach in CCMm may not be necessary, and the option of targeting only villages lacking a health centre or not located close to one should be considered – the logic behind CCMm being precisely “to reach users who cannot appropriately be served by the formal health centre” [25].

### Limits

The observational nature of this study and the absence of baseline measures do not permit inference of causality. Anticipating this, various strategies were adopted to increase internal validity: selection of a control site; repeated post-intervention measures; evaluation of implementation fidelity; and close follow-up of context [69]. While this study cannot pretend to be an evaluation of CCMm effects, results presented here concerning treatment-seeking actions, along with results presented elsewhere, shed light on the effectiveness of the Burkinabé programme [36,37]. The low number of sick children who sought treatment from CHWs was unexpected and limited the power of the analyses. This likely explains the large confidence intervals in the model and why previously identified determinants of treatment-seeking behaviour were not statistically significantly associated with the odds of consulting a CHW.

Because of the above-mentioned lack of power, child sickness episodes were analysed rather than febrile episodes. The facts that about 88% of sick children reported fever and that the proportion of children who visited a CHW was similar among those with or without fever supported this decision. Because of missing data (6%), the duration of sickness episodes was not integrated into the model, but analyses on the sub-sample with complete data showed that this variable was non-significant and did not change the coefficients of the other variables.

The proportion of caregivers stating that they do not know the CHW might be higher than in actuality because (1) information bias cannot be excluded – claiming not to know the CHW is a neutral answer that might have been given to avoid embarrassment; and (2) it is impossible to determine whether caregivers meant to say, “I don’t know of the CHW’s existence” or, “I don’t know the CHW personally”.

The external validity of this study is limited by the fact that it took place within a 20–km radius of the cities of Kaya and Zorgho, which are moderately sized cities. Treatment-seeking practices might have been different in villages more distant from the city, although none of the field visits or local informal interviews suggested this. Also, Euclidian distances between households and health centres were used, an approach that assumes households always visit the nearest health centres, failing to account for topographical barriers; other geographical approaches were inappropriate (i.e., drive time) or required unavailable data (i.e., network analyses) [70].

## Conclusions

This study evaluated treatment-seeking behaviour for sick children after the introduction of CCMm in Burkina Faso. It seems to be the longitudinal study examining a CCMm programme implemented in real-world conditions and at a national scale. The study shows that CHWs are rarely used and suggests that issues related to implementation fidelity, acceptability or feasibility have undermined the effectiveness of the programme in Burkina Faso. During the three-year survey period, treatment coverage of sick children by CHWs never exceeded 1% in urban areas and 10% in rural areas. The results of this study differ from those of previous evaluations of CCMm and show the importance of conducting evaluations under real-world conditions of implementation [71]. This study also confirms that distance to the nearest health centre and home visits paid by CHWs are statistically significant determinants of consulting a CHW.

The theory behind CCMm is to reduce the monetary and geographical barriers to ACT treatment in remote communities. In the Burkinabé context, it seems inappropriate to expand this strategy in urban areas. Barriers most certainly exist in urban areas as well, but CCMm did not help to reduce them. Several reasons have been advanced here to explain the lack of success of CCMm in rural areas. Arguably, one of the most important reason is that CCMm was not given sufficient consideration and funding to attain its ambitious objectives. Also, it is essential to evaluate pilot projects before considering scaling up an intervention such as CCMm to the national level. Such evaluations provide valuable information on feasibility and acceptability, as well as on requirements for adapting the strategy to the local context. Despite the general enthusiasm for pursuing Millennium Development Goals, policies recommended by international organizations should be carefully assessed under a country’s real-world conditions and adapted to local context as necessary.

While CHWs’ potential for improving child health is not questioned here [72], community case management strategies are not easy to implement and require measures to strengthen national health systems. Issues related to the current medicalization of CHWs in SSA have to be acknowledged and addressed [73].

## Additional file

Additional file 1:
**Use of CHW as first treatment-seeking action for sick children according the presence of fever or danger signs.**

## Abbreviations

ACT: Artemisinin-based combination therapy
CCMm: Community case management of malaria
CHW: Community health worker
GPS: Global positioning system
HDSS: Health and demographic surveillance system
PDA: Personal digital assistant
SSA: Sub-Saharan Africa
