# Supplementary material for: Utilization of community health workers for malaria treatment: results from a three-year panel study in the districts of Kaya and Zorgho, Burkina Faso
Source: Malar J. 2015 Feb 13;14:71. doi: 10.1186/s12936-015-0591-9 (PMC4329655; doi:10.1186/s12936-015-0591-9)
Supplement: Additional file 1: — Use of CHW as first treatment-seeking action for sick children according the presence of fever or danger signs. [file 12936_2015_591_MOESM1_ESM.pdf]

Additional file 1 Use of CHW as first treatment-seeking action for sick children according the presence of fever or danger signs.

Comparison of use of CHWs by sick children with vs. without fever

---

-> year = 2011

Key
frequency
column percentage

First used CHW	With fever? yes	no	Total
no	555	127	682
	96.19	98.45	96.60
yes	22	2	24
	3.81	1.55	3.40
Total	577	129	706
	100.00	100.00	100.00

Pearson chi2(1) = 1.6433 Pr = 0.200

-> year = 2012

Key
frequency
column percentage

First used CHW	With fever? yes	no	Total
no	644	130	774
	97.43	99.24	97.73
yes	17	1	18
	2.57	0.76	2.27
Total	661	131	792
	100.00	100.00	100.00

Pearson chi2(1) = 1.6100 Pr = 0.204

```
--------------------------------------------------------------------------------------

-> year = 2013

+-------------------+
| Key               |
|-------------------|
|     frequency     |
| column percentage |
+-------------------+

First used |       With fever?     |
CHW        |       yes        no   |     Total
-----------+----------------------+----------
        no |       573         82 |       655 
           |     94.55      96.47 |     94.79 
-----------+----------------------+----------
       yes |        33          3 |        36 
           |      5.45       3.53 |      5.21 
-----------+----------------------+----------
     Total |       606         85 |       691 
           |    100.00     100.00 |    100.00 

          Pearson chi2(1) =   0.5542   Pr = 0.457
```

Comparison of use of CHWs by sick children with vs. without danger signs

```
--------------------------------------------------------------------------------------

-> year = 2011

+-------------------+
| Key               |
|-------------------|
|     frequency     |
| column percentage |
+-------------------+

First used |  With danger signs?  |
CHW        |       yes        no  |     Total
-----------+----------------------+----------
        no |       500        182 |       682 
           |     96.34      97.33 |     96.60 
-----------+----------------------+----------
       yes |        19          5 |        24 
           |      3.66       2.67 |      3.40 
-----------+----------------------+----------
     Total |       519        187 |       706 
           |    100.00     100.00 |    100.00 

          Pearson chi2(1) =   0.4079   Pr = 0.523
```

```
--------------------------------------------------------------------------------------------

-> year = 2012

+-------------------+
| Key               |
|-------------------|
|     frequency     |
| column percentage |
+-------------------+

First used |  With danger signs?  |
CHW        |       yes        no  |     Total
-----------+----------------------+----------
        no |       607       167 |       774 
           |     98.06     96.53 |     97.73 
-----------+----------------------+----------
       yes |        12         6 |        18 
           |      1.94      3.47 |      2.27 
-----------+----------------------+----------
     Total |       619       173 |       792 
           |    100.00    100.00 |    100.00 

          Pearson chi2(1) =   1.4243   Pr = 0.233

--------------------------------------------------------------------------------------------

-> year = 2013

+-------------------+
| Key               |
|-------------------|
|     frequency     |
| column percentage |
+-------------------+

First used |  With danger signs?  |
CHW        |       yes        no  |     Total
-----------+----------------------+----------
        no |       490       160 |       650 
           |     94.41     96.39 |     94.89 
-----------+----------------------+----------
       yes |        29         6 |        35 
           |      5.59      3.61 |      5.11 
-----------+----------------------+----------
     Total |       519       166 |       685 
           |    100.00    100.00 |    100.00 

          Pearson chi2(1) =   1.0100   Pr = 0.315
```
